# Supplementary material for: Supramolecular coupling of cylindrical micelles following seeded-growth
Source: Nat Commun. 2026 Feb 26;17:3247. doi: 10.1038/s41467-026-69785-3 (PMC13062008; doi:10.1038/s41467-026-69785-3)
Supplement: Supplementary file 1 — Supplementary Information [file 41467_2026_69785_MOESM1_ESM.pdf]

Supplementary Information

Supramolecular coupling of cylindrical micelles following seeded-growth

Wenhao Gao[1†], Kaiwen Sun[1†], Xiaosong Wang[2*], Liang Gao[1, 3*], Jiaping Lin[1, 3, 4, 5*], Chengyan Zhang[1] & Chunhua Cai[1, 3]

[1] School of Materials Science and Engineering, East China University of Science and Technology, Shanghai 200237, China

[2] Department of Chemistry, Waterloo Institute for Nanotechnology, University of Waterloo, Waterloo N2L 3G1, Canada

[3] Shanghai Key Laboratory of Advanced Polymeric Materials, East China University of Science and Technology, Shanghai 200237, China

[4] Key Laboratory for Ultrafine Materials of Ministry of Education, East China University of Science and Technology, Shanghai 200237, China

[5] Frontiers Science Center for Materiobiology and Dynamic Chemistry, East China University of Science and Technology, Shanghai 200237, China

---

[†] These authors contributed equally.

✉e-mail: xiaosong.wang@uwaterloo.ca; lianggao@ecust.edu.cn; jlin@ecust.edu.cn

Contents

1. Experiments .... **SI 3**

1.1 Polymer synthesis .... **SI 3**

1.2 Preparation method of seed micelles and segmented nanowires .... **SI 6**

1.3 Characterization methods of assemblies .... **SI 8**

1.4 Turbidity measurements of the PBLG-*b*-PNIPAM diblock copolymers .... **SI 10**

1.5 Distribution of the contour length of the seed micelles .... **SI 11**

1.6 Distinction and statistics of seed and grown regions .... **SI 12**

1.7 Rationale for choosing the $PBLG_{237}$-*b*-$PNIPAM_{118}$ system .... **SI 14**

1.8 Effect of unimer-to-seed feed ratios .... **SI 16**

1.9 Control experiment of stepwise addition of seeds .... **SI 19**

1.10 Characterization of small aggregates in solution without seeds .... **SI 20**

1.11 Temporal evolution of morphologies during growth and coupling .... **SI 22**

1.12 Effect of the methanol content on the growth of the segmented nanowires .... **SI 25**

1.13 Effect of the solvent types on the growth of the segmented nanowires .... **SI 27**

1.14 Effect of the incubation temperature .... **SI 32**

2. Theoretical Simulations .... **SI 33**

2.1 Brownian dynamics simulation method .... **SI 33**

2.2 Coarse-grained model and parameter settings .... **SI 36**

2.3 Growth and coupling behavior of cylindrical micelles .... **SI 39**

2.4 Effect of interaction parameter on growth and coupling behaviors .... **SI 42**

References .... **SI 44**

1. Experiments

1.1 Polymer synthesis

Reagents and materials. L-Glutamic acid γ-benzyl ester (BLG), ethyl acetate, petroleum ether, sodium chloride, and anhydrous magnesium sulfate were purchased from Adamas-beta. Epoxypropane and triphosgene were purchased from TCI. *N*-isopropylacrylamide (NIPAM, 98%, TCI) was purified by recrystallization from n-hexane. 2,2-Azobis(2-Methylpropionitrile) (AIBN, 98%, TCI) was recrystallized from absolute methanol. 2-Aminoethanethiol Hydrochloride (AET·HCl, 95%, TCI) was used without further purification. Methanol (MeOH), Ethanol (EtOH), Tetrahydrofuran (THF), *N*, *N'*-Dimethyl Formamide (DMF), and 1,4-Dioxane (Diox) of analytical grade were purchased from Adamas-beta. The dialysis bag (14000 and 3500 molecular weight cutoff) was obtained from Serva Electrophoresis GmbH. Deionized water was prepared in a Direct-Q 8 UV Remote Water System to a level of 18 MΩ·cm resistance.

Synthesis of PBLG-*b*-PNIPAM diblock copolymers. The monomer γ-Benzyl-L-glutamate-N-carboxyanhydride (BLG-NCA) was synthesized by the triphosgene method as follows [S1-S3]. Specifically, L-Glutamic acid γ-benzyl ester (BLG, 15 g), epoxypropane (20 mL), and triphosgene (11.7 g) were mixed in 225 mL of tetrahydrofuran. After stirring for 2 h at room temperature, the reaction was quenched with deionized water. The reaction mixture was then sequentially washed with ethyl acetate (150 mL) and saturated brine (200 mL). The organic phase was dried over anhydrous magnesium sulfate. Following the removal of the organic

solvent under vacuum, the crude residue was purified by recrystallization in petroleum ether. The synthesis method of the initiator PNIPAM-NH_2 is as follows [S4, S5]. AIBN was used as the initiator and AET·HCl as the chain transfer agent to synthesize monoamine-terminated PNIPAM (PNIPAM-NH_2) by free radical polymerization of NIPAM in methanol at 60 °C. The degree of polymerization (DP) and polydispersity index ($Đ$) for the resultant PNIPAM-NH_2 were 118 and 1.35, respectively, as measured by the gel permeation chromatography analysis using DMF as eluent and calibrated with polymethyl methacrylate standards. Poly(γ-benzyl-L-glutamate)-*block*-poly(N-isopropylacrylamide) (PBLG-*b*-PNIPAM) diblock copolymers (BCPs) were synthesized by ring-opening polymerization of BLG-NCA initiated by PNIPAM-NH_2 macroinitiator with anhydrous 1,4-dioxane as the solvent. The reaction was performed in a flame-dried reaction eggplant flask under a dry nitrogen atmosphere at 15 °C for 3 days. At the end of the polymerization, the viscous reaction mixture was poured into anhydrous methanol. The resulting products were dried under vacuum and then purified twice by repeatedly precipitating a chloroform solution into a large volume of anhydrous methanol.

The PBLG-*b*-PNIPAM diblock copolymers were characterized by gel permeation chromatography (GPC, PL GPC-50 plus, Varian) and nuclear magnetic resonance (^{1}H NMR, Avance 600 MHz, Bruker). As shown in Supplementary Figure 1a, the GPC trace displays a monomodal symmetric distribution with a $Đ$ value of 1.40. Supplementary Figure 1b shows the typical ^{1}H NMR spectrum of PBLG-*b*-PNIPAM diblock copolymers in $CDCl_3$ with 15 vol% TFA-d. Since the number average molecular weight (M_n) of the PNIPAM block is 1.3×10^4

g/mol (the DP value was calculated to be 118), the M_n of the PBLG block can be calculated by the peak intensity ratio of the methylene proton signal (5.1 ppm) of PBLG to the methylene proton signal (3.6 ppm) of PNIPAM. The M_n of the PBLG-*b*-PNIPAM diblock copolymers is calculated as 6.5×10^4 g/mol. Because the molecular weight of PBLG repeat units is 219 g/mol, the DP value of PBLG is 237. We then denote the synthesized diblock copolymer as $PBLG_{237}$-*b*-$PNIPAM_{118}$.

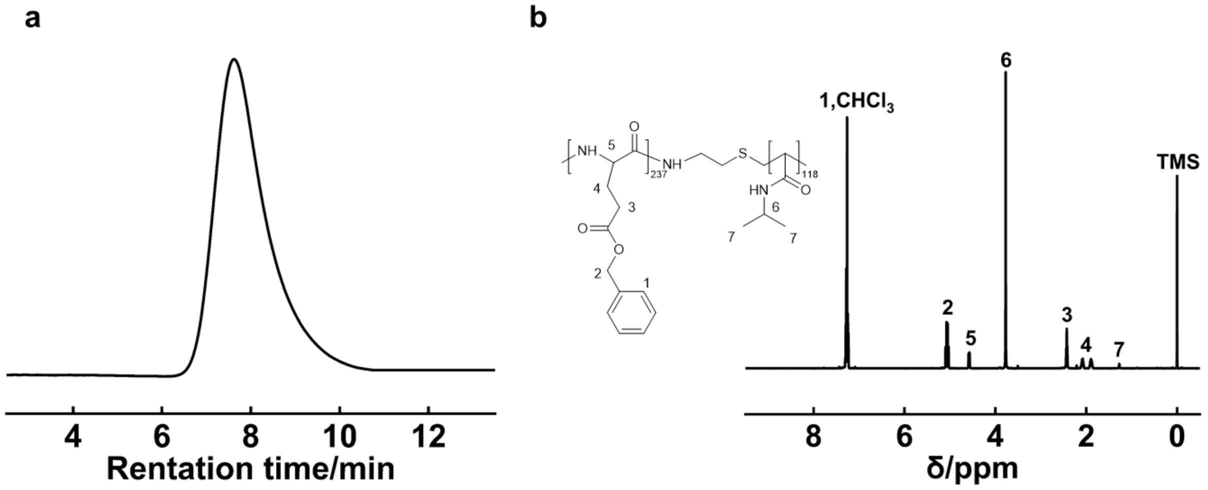

Supplementary Figure 1. Characterizations of the diblock copolymer. (a) GPC and (b) ^{1}H NMR characterization results of PBLG-*b*-PNIPAM copolymers. GPC measurement was performed at 50 °C with DMF as the eluent solution. ^{1}H NMR measurement was recorded using an Avance 600 instrument (operating at 600 MHz) with $CDCl_3$ with 15 vol% TFA-d as the solvent.

1.2 Preparation method of seed micelles and segmented nanowires

The seed micelles were prepared by a selective precipitation method. Firstly, 2.4 mL of methanol was added to 2.0 mL of PBLG-*b*-PNIPAM copolymers THF solution (copolymers concentration of 0.08 g/L) in 3 min with vigorous stirring at 30 °C. The transparent solution gradually became tinted blue with the addition of methanol, which indicated the formation of aggregates. Then, a solution of PBLG-*b*-PNIPAM copolymers dissolved in DMF (2.0 g/L, 40 μL) was added to the seed solution (4.4 mL, THF/methanol, v/v, 45.5/54.5, $C_{seed} = 3.6\times10^{-2}$ g/L). The experimental schematic is shown in Supplementary Figure 2.

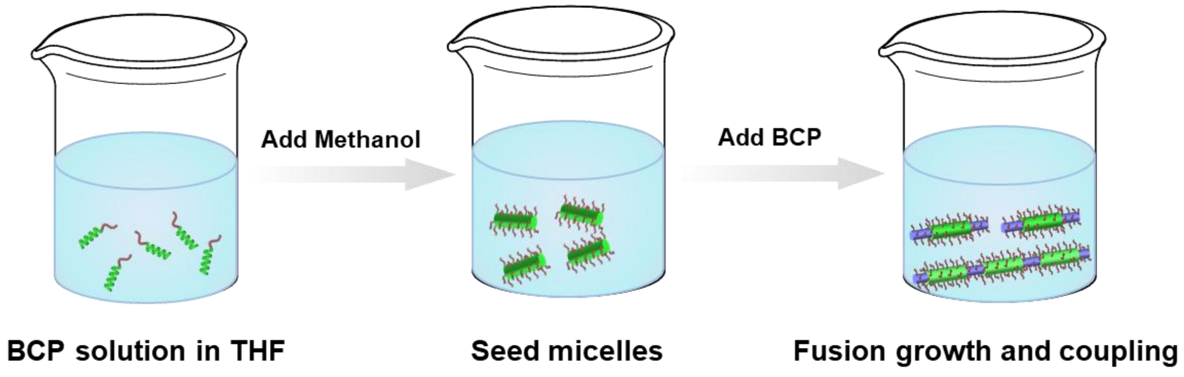

Supplementary Figure 2. Schematic of the preparation of seed micelles, and subsequent fusion growth and coupling.

The mixed solution was aged for various incubation times. Before the characterization of aggregate structures, the incubated solutions were dialyzed against deionized water for at least 3 days to ensure the organic solvent had been removed. Notably, DMF, relative to THF, has a better solvation power for PBLG-*b*-PNIPAM. We choose THF for seed preparation because DMF leads to irregular aggregates. For seeded growth, we need to prepare a highly concentrated

polymer solution to minimize the amount of good solvents in the growth system, so DMF is used. The small amounts of DMF do not affect either micelle growth or coupling; instead, it dissolves well the added copolymer during feeding.

1.3 Characterization methods of assemblies

Characterization techniques of morphology. The morphologies of the seeds and cylindrical micelles were observed by transmission electron microscopy (TEM, JEM-1400, JEOL, 100 kV) and atomic force microscopy (AFM, XE-100, Park Systems, noncontact mode). Samples were prepared by placing a drop of solution on a copper grid coated with carbon film for the TEM testing or a cleaned silicon wafer for AFM testing, and then dried at room temperature.

Dynamic and static light scattering. Dynamic and static light scattering (DLS) measurements were performed by an LLS spectrometer (ALV/CGS-5022) equipped with an ALV-High QE APD detector and an ALV-5000 digital correlator using a He-Ne laser (wavelength λ = 632.8 nm) as the light source. All light scattering measurements were carried out at a constant temperature of 30 °C. Before measurements, all sample solutions were filtered through a polytetrafluoroethylene filter.

Calculation of the aggregate size. The length of seeds and cylindrical micelles was estimated from the TEM images using the Image-Pro Plus software. More than 200 objects were processed to calculate the contour length. From the statistical results, histograms were constructed, and the number-average length (L_n), the weight-average length (L_w), and the polydispersity index (PDI) were estimated using the following equations, respectively, where N is the number of micelles.

$$L_n = \frac{\sum_{i=1}^{n} N_i L_i}{\sum_{i=1}^{n} N_i} \quad \text{(S-1)}$$

$$L_w = \frac{\sum_{i=1}^{n} N_i L_i^2}{\sum_{i=1}^{n} N_i L_i} \quad \text{(S-2)}$$

$$PDI = \frac{L_w}{L_n} \quad \text{(S-3)}$$

Wide-angle X-ray scattering. Synchrotron radiation Wide-angle X-ray scattering (WAXS) measurement was performed at beamline BL16B1 of Shanghai Synchrotron Radiation Facility. To obtain the powder sample for the WAXS measurement, the micelle solutions of seeds, small aggregates, and cylindrical micelles were prepared and dried by the vacuum freeze dryer. The final products (about 10 mg for each sample) were collected for characterizations. The powder sample dried from micelle solution was wrapped with a special adhesive tape and placed on the sample table for testing. In the *in situ* WAXS measurement, the micelle solution was injected into a sample pool for testing, where the other characterization details of WAXS were similar to those of the powder sample. The distance of the sample to the detector for WAXS was calibrated to be 192 mm, and the wavelength of the X-ray is 0.124 nm (E = 10 keV). To probe the structure of the samples, the exposure time was set to 60s. The two-dimensional WAXS pattern was collected with a Mar165 CCD detector (2048×2048 pixels with a pixel size of 80 μm). The WAXS data were analyzed with the Fit2D software from the European Synchrotron Radiation Facility. The d-spacing values were calculated from Bragg's Law, $d = 2\pi/q$.

1.4 Turbidity measurements of the PBLG-*b*-PNIPAM diblock copolymers

The critical methanol content (CMC) of the polymers, an indication of the onset of their aggregation, is measured by recording abrupt changes in turbidity during methanol addition. The CMC was measured at a wavelength of 300 nm with a UV-Vis spectrophotometer (UV-2550 Shimadzu). The polymer solution concentration was 0.08 g/L in THF, the initial volume was 2 mL, and the volume of each addition of methanol was 10 μL. As shown in Supplementary Figure 3, the critical methanol content of $PBLG_{237}$-*b*-$PNIPAM_{118}$ is 42.5 vol%.

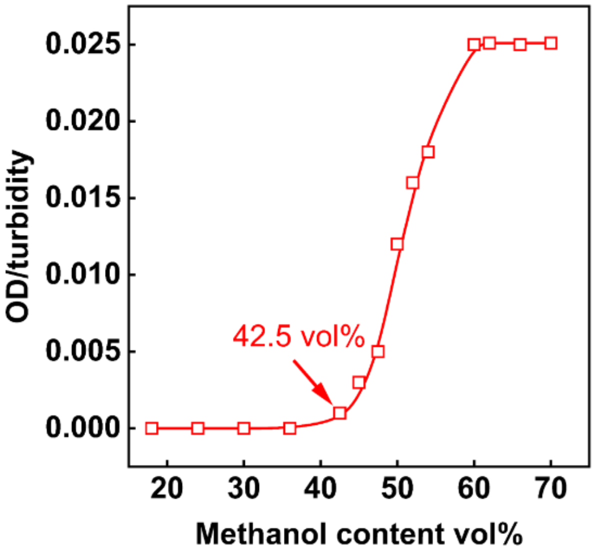

Supplementary Figure 3. Turbidity curves of $PBLG_{237}$-*b*-$PNIPAM_{118}$ block copolymers as a function of methanol content in solution.

1.5 Distribution of the contour length of the seed micelles

The length of the seed micelles was estimated from TEM images using Image-Pro Plus software. For statistical length analysis, the contour lengths of over 200 objects were measured. Based on the statistical results, the number-average length (L_n) and weight-average length (L_w) were calculated to be 490 nm and 540 nm, respectively, with a polydispersity index (PDI) of 1.10. The contour length distribution of the seed micelles is presented in Supplementary Figure 4.

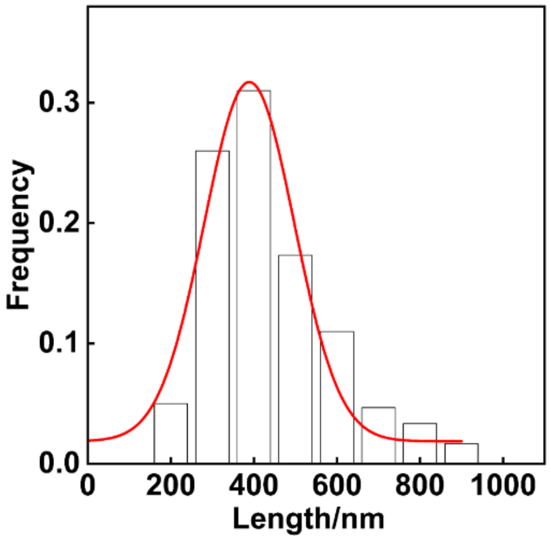

Supplementary Figure 4. Distribution of the contour length of the seed micelles.

1.6 Distinction and statistics of seed and grown regions

From the high-magnification TEM image of the segmented nanowire (Supplementary Figure 5), it is clearly observed that there are significant differences in the widths of the seed and grown regions. We analyzed more than 200 TEM images using the Image-Pro Plus software and automatically identified the seed and grown segments per nanowire based on the difference in width.

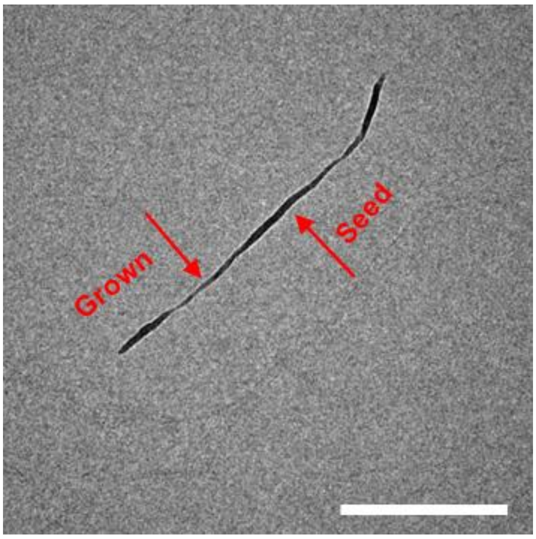

Supplementary Figure 5. High-magnification TEM image of segmented nanowires. Scale bars: 1 μm.

In addition, as shown in Supplementary Equations (4) and (5), the segmented nanowires are divided into seed parts($N^{\mathrm{SDDE}}L^{\mathrm{SEED}}$, the contributions of the total length of the seed parts to the nanowire length) and grown parts (L^{GROWN}, the contributions of the total length of the grown parts to the nanowire length).

$$L = N^{SEED}L^{SEED} + L^{GROWN} \tag{S-4}$$

$$N^{SEED} = \sum_{i=1}^{n} in_i / \sum_{i=1}^{n} n_i \quad \text{(S-5)}$$

where L is the total length of segmented nanowires, L^{SEED} is the number-average length of the initial seed, N^{SEED} is the average number of seeds in the segmented nanowire, and n_{i} is the number of segmented nanowires with the seed number of i. Therefore, the $N^{\mathrm{SEED}}L^{\mathrm{SEED}}$ is the average length of seed parts. In addition, due to the coupling occurring at the ends of the grown cylindrical micelles, L^{GROWN} is used to represent the number-average length of the grown parts.

1.7 Rationale for choosing the $PBLG_{237}$-*b*-$PNIPAM_{118}$ system

Complementing experiments with theoretical simulations, the general rules for achieving controlled LC-driven coupling can be summarized as: **1)** LC cores should rearrange more slowly during coupling than during fusion, requiring extended twisting and alignment. **2)** The solubility parameters of the LC rod block and coil block should be sufficiently close to allow this rearrangement. **3)** A moderate block ratio (*e.g.*, ~2:1 for $PBLG_{237}$-*b*-$PNIPAM_{118}$) is needed to balance packing frustration and flexibility. **4)** Optimized solvent conditions should allow tunable LC ordering. When these conditions are met, the hierarchical segmented nanowires can form, demonstrating the mechanism's generality.

We also tested other LC-containing block copolymers. For example, $PBLG_{226}$-*b*-PEG_{112} failed to produce segmented nanowires in either THF/MeOH/DMF or Diox/MeOH/DMF, yielding only irregular fibers or cylinders (Supplementary Figure 6). This is consistent with the design rules: PEG is far more hydrophilic, and its solubility mismatch with PBLG prevents the necessary LC rearrangement.

In contrast, PBLG-*b*-PNIPAM satisfies all requirements. PBLG and PNIPAM have closely matched Hansen solubility parameters, and the THF/MeOH/DMF solvent combination provides the right balance for LC ordering and rearrangement. Therefore, the choice of $PBLG_{237}$-*b*-$PNIPAM_{118}$ is not arbitrary — it is essential for enabling the controllable LC-driven coupling process.

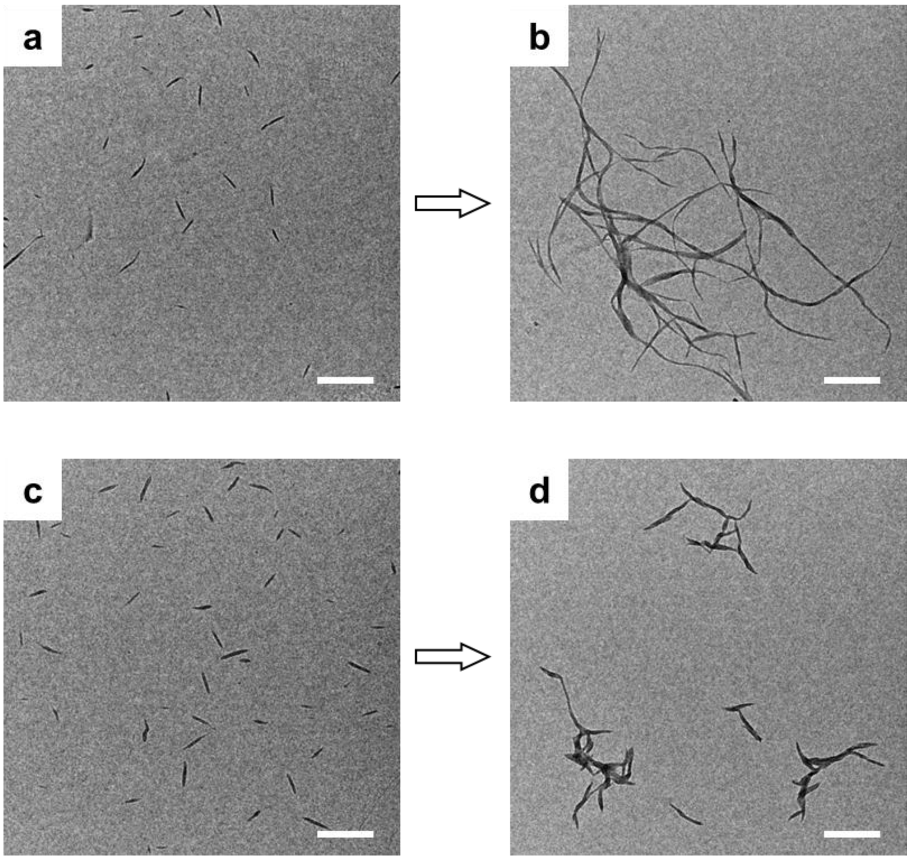

Supplementary Figure 6. Nanostructures formed by the $PBLG_{226}$-*b*-PEG_{112} copolymers in various solvent. (a,c) TEM images of seed micelles prepared by $PBLG_{226}$-*b*-PEG_{112} copolymers in (a) THF/MeOH and (c) Diox/MeOH solutions. (b,d) TEM images of aggregates after feeding PBLG-*b*-PEG copolymers and aging at 30 °C for 48 hours in (b) THF/MeOH/DMF and (d) Diox/MeOH/DMF solutions. Scale bars: 1 μm.

1.8 Effect of unimer-to-seed feed ratios

Here, the feed ratio (*i.e.*, unimer-to-seed ratio) is defined as the ratio of the added copolymer mass (m_{unimer}) to the initial copolymer mass (m_{seed}). To change the feed ratio, the PBLG-*b*-PNIPAM unimer solution of various volumes (40 μL, 80 μL, 120 μL, 160 μL, 200 μL, 240 μL) with the copolymer concentration of 2 g/L in DMF was added to the dilute solution of seeds (4.4 mL, $C_{\text{seed}} = 1.8\times10^{-2}$ g/L, THF/methanol = 45.5/54.5, v/v), respectively. The mixtures were aged for various incubation times to examine the temporal evolution of morphologies.

Supplementary Figure 7a-b indicates that fusion growth and coupling occur when the feed ratio is 2.0, resulting in the formation of segmented nanowires. Supplementary Figure 7c presents the temporal variations in the length of segmented nanowires at various unimer-to-seed ratios. Supplementary Figure 7d shows the temporal variations of the seed number N^{SEED} for the segmented nanowires formed at various unimer-to-seed ratios. Notably, Supplementary Figure 7c shows the time-dependent evolution of SEGMENTED lengths during the growth process, while Fig. 1i in the main text presents the final SEGMENTED lengths measured after 72 hours at various unimer-to-seed ratios. When we compare only the data collected under the same conditions (72 hours and identical unimer-to-seed ratios), the values in Fig. 1i and Supplementary Figure 7c agree very well. For example, at ratios of 2, 3, and 5, the final lengths are 2.63 ± 0.13 μm, 3.12 ± 0.20 μm, and 4.37 ± 0.22 μm in Fig. 1i, which match the corresponding values of 2.60 ± 0.13 μm, 3.13 ± 0.18 μm, and 4.28 ± 0.12 μm in Supplementary Figure 7c. The small differences fall within normal statistical variations.

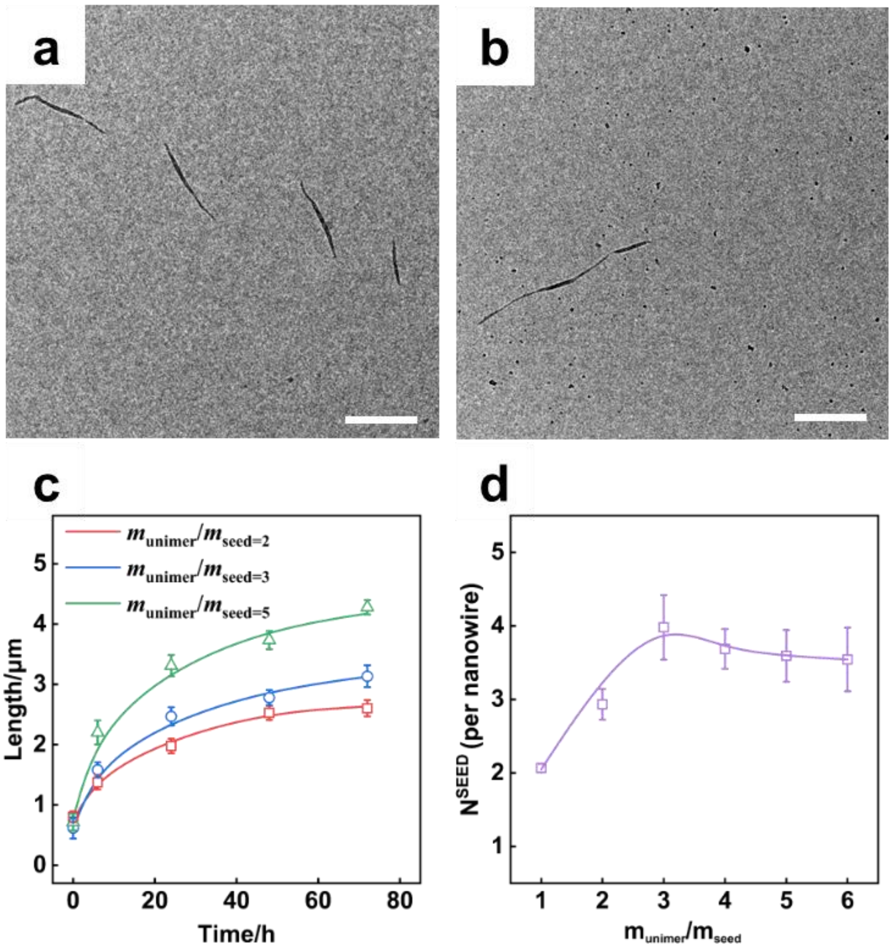

Supplementary Figure 7. Effect of unimer-to-seed feed ratios. (a-b) TEM images of the assembly after incubating for (a) 6 hours and (b) 48 hours when the feed ratio is 2.0. Scale bars: 1μm. (c) Temporal variations of the length of segmented nanowires at various unimer-to-seed ratios. Error bars represent mean ± standard deviation, $n \geq 200$. (d) Temporal variations of the seed number N^{SEED} for the segmented nanowires formed at various unimer-to-seed ratios. Error bars represent mean ± standard deviation, $n \geq 200$.

The curves of Supplementary Figure 7c show that the length of micelles rapidly increases during the initial stage, slows down in the later stage, and eventually reaches a plateau. The final equilibrium of the curves can be attributed to the increase in the length of the segmented nanowires. This increase significantly affects the mobility and diffusion ability of micelles, which are crucial for the colloidal assembly process. In addition, as the feed ratio increases, the

length of the grown part is longer in the initial stage, leading to longer final segmented nanowires. This is because a higher feed ratio results in the formation of more small aggregates in the solution, which increases the probability of collisions and fusion with seeds.

1.9 Control experiment of stepwise addition of seeds

Here, we performed a control experiment regarding stepwise seed addition. Adding more seed solution after the first feeding and incubating for 24 hours, it can still produce segmented nanowires, but increases the average number of seeds per nanowire from ~3.3 to ~4.5 (Supplementary Figure 8). This demonstrates that multiple feedings can adjust the hierarchical structure, yet the underlying assembly still proceeds through the exact LC-driven end-to-end coupling mechanism.

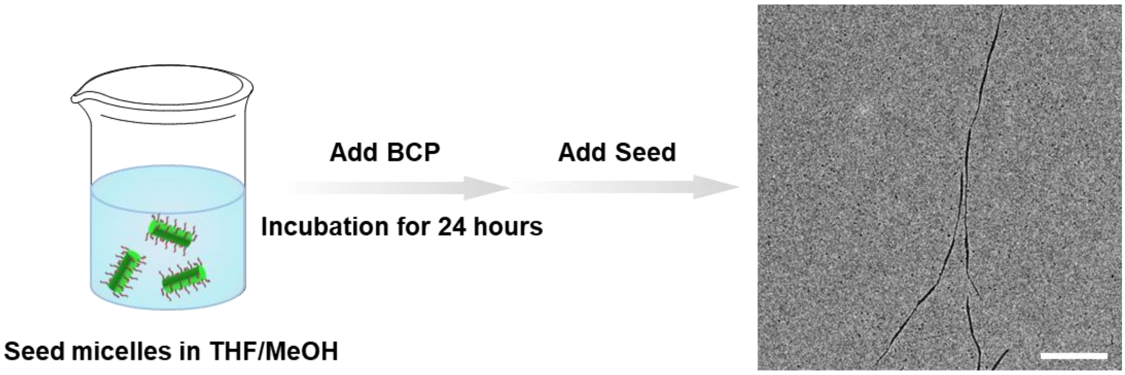

Supplementary Figure 8. Schematic illustration and representative morphology of segmented nanowires prepared by stepwise addition of seed. Scale bars: 1μm.

1.10 Characterization of small aggregates in solution without seeds

To further investigate the fusion growth mechanism, we conducted experiments focusing on small aggregates assembled from the copolymers. Here, the copolymers (2.0 g/L in DMF, 40 μL) were introduced into a mixed solvent of methanol and THF (4.4 mL; THF/methanol, v/v, 45.5/54.5) without the presence of seed micelles. The temporal evolution of the aggregates was analyzed using TEM. As shown in Supplementary Figure 9a-b, the copolymers formed small aggregates, but their size exhibited minimal change with increasing incubation time.

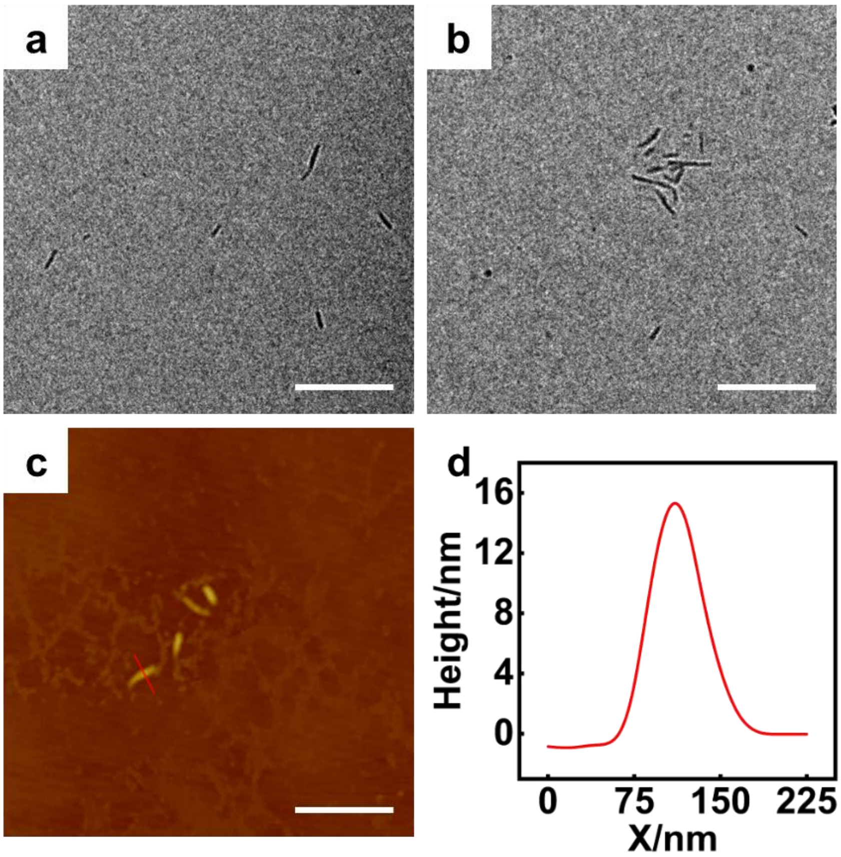

Supplementary Figure 9. Characterizations of the formed small aggregates. (a-b) TEM images of the aggregates through adding unimers into the mixture solution: (a) 0 h, (b) 48h. (c) AFM image of the aggregates prepared in the mixture solution. (d) Height profile along the selected region in (c). Scale bars: 1μm.

The structure of these small aggregates was further characterized using AFM. In Supplementary Figure 9c-d, the height of the aggregates was measured to be approximately 15 nm. The results indicate that the height of the small aggregates in the solution is smaller than the seeds in the solution. This is due to that the small aggregates are assembled in different solvent conditions, resulting in a poor ordering of chains in these small aggregates.

1.11 Temporal evolution of morphologies during growth and coupling

Here, the TEM images of aggregates corresponding to various time points in the DLS curves (Supplementary Figure 10a, *i.e.*, Fig. 2c in the main text) were provided. Small aggregates are visible in Supplementary Figure 10b, matching the small peak in the DLS data. As incubation continues, this peak gradually weakens and disappears (red and blue curves in Supplementary Figure 10a). TEM images confirm that these aggregates fuse onto the ends of the seed micelles during growth (Supplementary Figure 10c, 6 h; Supplementary Figure 10d, 24 h).

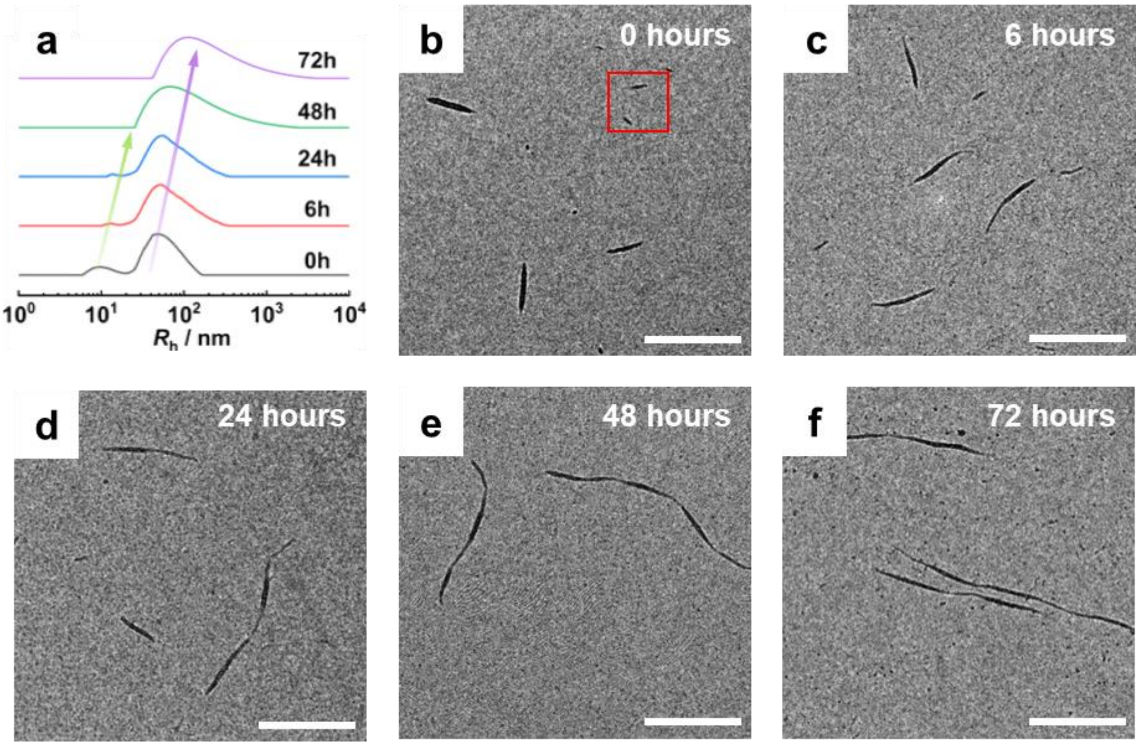

Supplementary Figure 10. Temporal evolution of morphologies during growth and coupling. (a) Temporal evolution of the R_h of the seed solution containing added copolymer unimers. (b-f) TEM images corresponding to various time points in the DLS curves of (b) 0 hours, (c) 6 hours, (d) 24 hours, (e) 48 hours, and (f) 72 hours, respectively. Scale bars: 1 μm.

After 24 hours, end-to-end coupling becomes dominant, producing segmented nanowires (Supplementary Figure 10e, 48 h; Supplementary Figure 10f, 72 h). At this stage, R_h increases, and the DLS peaks broaden (green and purple curves). Overall, the TEM observations are fully consistent with the DLS results.

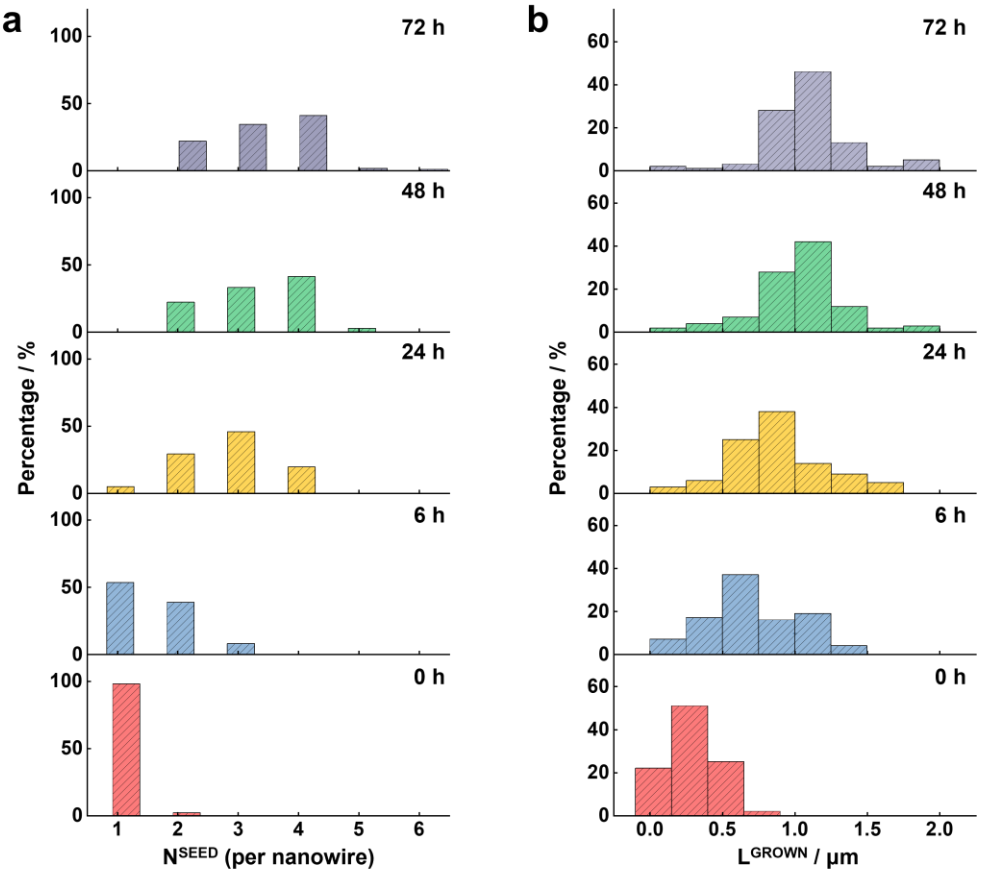

Supplementary Figure 11. Temporal evolution of N^{SEED} and L^{GROWN} during growth and coupling. Temporal variations of (a) the seed number N^{SEED} and (b) the L^{GROWN} for the segmented nanowires formed at methanol contents of 54.0 vol%.

To provide statistically meaningful evidence, we quantified the temporal distributions of seed number (N^{SEED}) and grown length (L^{GROWN}) for the 54.0 vol% MeOH condition (Supplementary Figure 11). Early in incubation (<6 h), most structures contain only 1–2 seeds,

reflecting fusion growth. By 24 h, N^{SEED} increases to 3–4, and by 48 h it stabilizes at ~4, indicating that nearly all initial seeds have participated in coupling, with no unreacted ends remaining. L^{GROWN} also increases rapidly within 6 h and narrows with time, confirming a transition from growth to controlled coupling.

1.12 Effect of the methanol content on the growth of the segmented nanowires

The effect of methanol content on the growth of the segmented nanowires was investigated. Various volumes of methanol were added to the seed solution, followed by the addition of polymer unimers. The mixture was then aged for 48 hours at 30 °C. The heights of the seed parts and the grown parts of the segmented nanowires formed at different methanol contents were measured. As shown in Supplementary Figure 12, the height of the grown parts or seed parts remained nearly unchanged with the addition of methanol.

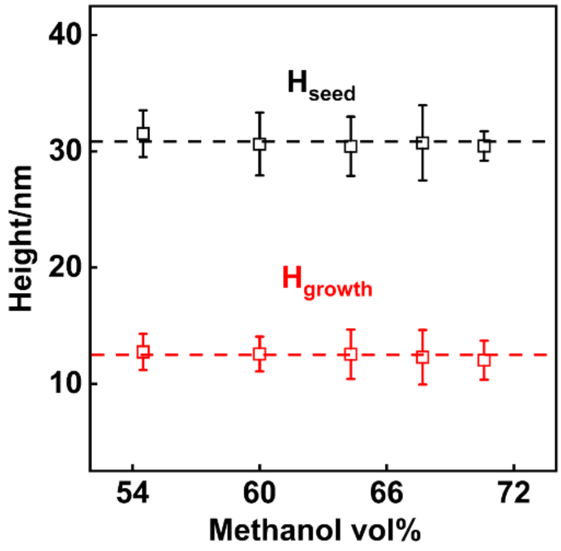

Supplementary Figure 12. Plots of the contour height of the seed region and growth region versus the ratio of Methanol. Error bars represent mean ± standard deviation, $n \geq 100$.

Supplementary Figure 13a shows the temporal variations of the seed number N^{SEED} for the segmented nanowires formed at various methanol contents. The statistical results indicate that the value of N^{SEED} decreases progressively with the increase in methanol content.

Supplementary Figure 13b shows the temporal variations of grown parts (L^{GROWN}) at various methanol contents. As can be seen, with the increase of methanol content, the length of the grown parts significantly decreases. The result indicates that the seeds growing through the fusion of small aggregates became difficult.

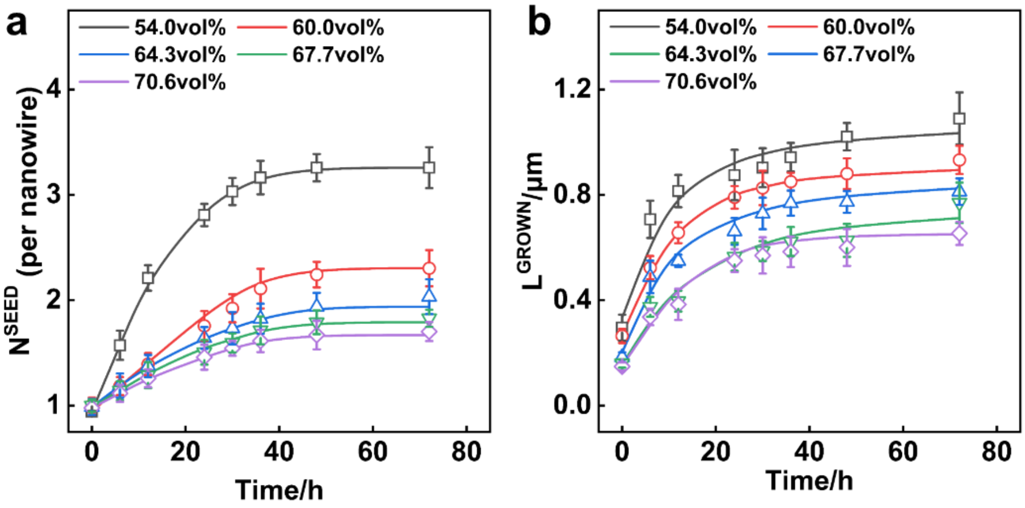

Supplementary Figure 13. Effect of the methanol content on the formation of the segmented nanowires. (a) Temporal variations of the seed number N^{SEED} for the segmented nanowires formed at various methanol contents. Error bars represent mean ± standard deviation, $n \geq 200$. (b) Temporal variations of the length L^{GROWN} of grown parts at various methanol contents. Error bars represent mean ± standard deviation, $n \geq 200$.

1.13 Effect of the solvent types on the growth of the segmented nanowires

To explore the effect of the solvent types on the growth of the segmented nanowires, we prepared seed micelles in different initial common solvents or selective solvents. In the first case, the common solvent THF was replaced by dioxane, while in the latter case, the selective solvent methanol was replaced by ethanol.

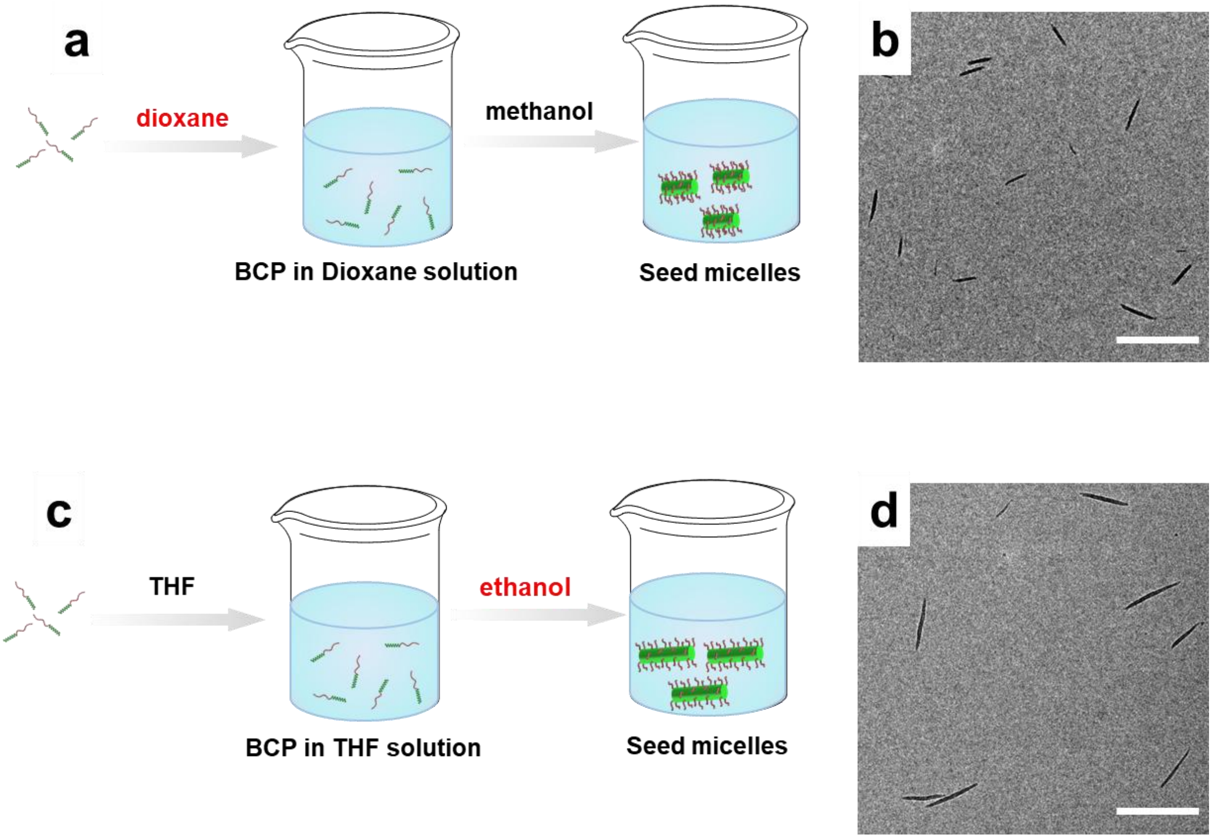

Supplementary Figure 14. Seed micelles formed in different solvents. (a) Schematic of the preparation of seed micelles in Diox/MeOH mixture solution. (b) TEM image of the seed micelles prepared in Diox/MeOH mixture solution. (c) Schematic of the preparation of seed micelles in THF/EtOH mixture solution. (d) TEM image of seed micelles prepared in THF/EtOH mixture solution. Scale bars: 1μm.

Supplementary Figure 14a-b and Supplementary Figure 14c-d show the preparation methods and the TEM images of seed micelles formed in Diox/MeOH and THF/EtOH mixture solutions, respectively. In the Diox/MeOH system, the obtained seeds have lengths of ~385 nm (Supplementary Figure 14b). While the seeds prepared in the THF/EtOH system have lengths of ~760 nm (Supplementary Figure 14d). Compared to the THF/EtOH and the THF/MeOH mixture solution, the length of the seed prepared in Diox/MeOH was shorter. Subsequently, incubation experiments of seed micelles prepared in different solvents were carried out. The incubation results are shown in Supplementary Figure 15.

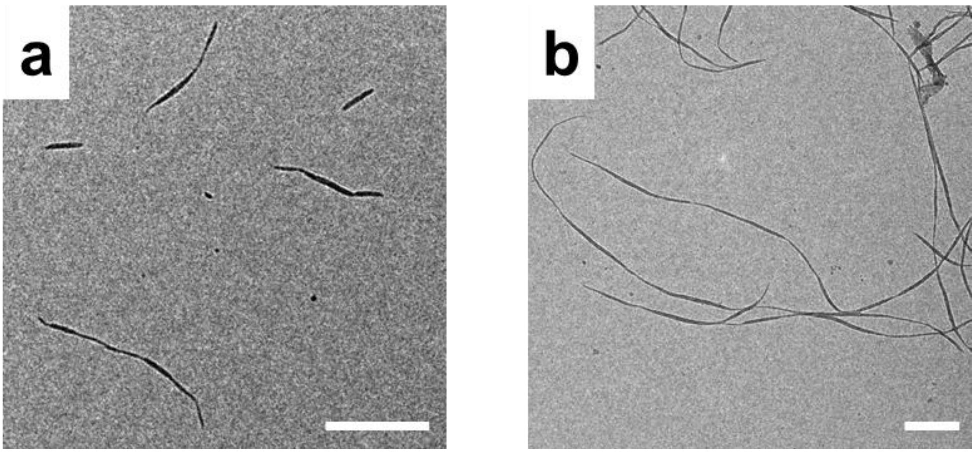

Supplementary Figure 15. Effect of the solvent types on the formation of the segmented nanowires. (a) TEM image of the segmented nanowires prepared in Diox/MeOH/DMF mixture solution after aging at 30 °C for 48 hours. (b) TEM image of the segmented nanowires prepared in THF/EtOH/DMF mixture solution after aging at 30 °C for 48 hours. Scale bars: 1μm.

The WAXS spectrum of the segmented nanowires under different solvent conditions is shown in Supplementary Figure 16. The results show that when ethanol is used as the selective solvent, the micelle core exhibits poor ordering due to weak interactions between PBLG blocks,

resulting in easier chain rearrangements. In contrast, when dioxane is used as the common solvent, the ordering of PBLG blocks in the micelle core improves due to enhanced solvation effects.

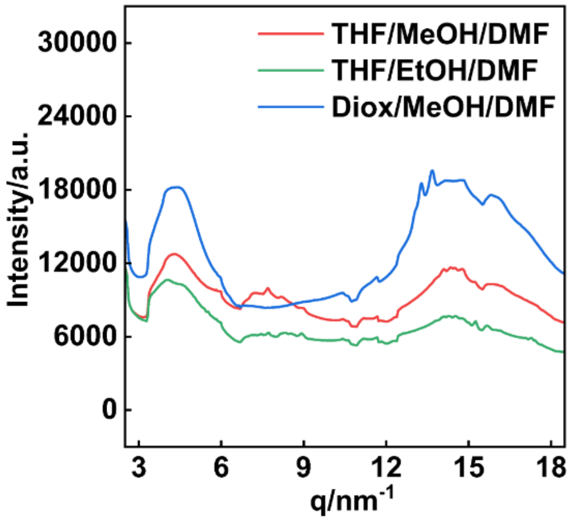

Supplementary Figure 16. WAXS spectra of the segmented nanowires in different solvents.

In addition, we prepared seed micelles under Diox/EtOH conditions and examined the growth and coupling behaviors of micelles in this solvent condition, where the solvent ratio, polymer concentration, and temperatures remain unchanged. At the 45.5/54.5 of Diox/EtOH in volume, the obtained seeds have lengths of ~550 nm (Supplementary Figure 17a). Subsequently, DMF solutions of the block copolymer (2.0 g/L, 40 μL) were added to these seed solutions for growth. After incubation for 48 hours, the segmented nanowires can be observed in the Diox/EtOH/DMF system (Supplementary Figure 17b). We tracked the variation of the number of seeds (N^{SEED}) per nanowire over time, and the results are shown in Supplementary Figure 17c. The N^{SEED} in the Diox/EtOH/DMF system reaches 3.8 (see the solid curve in

Supplementary Figure 17c). In addition, the total length of grown parts (L^{GROWN}) reaches ~1800 nm. It was found that the micelle growth and coupling in the Diox/EtOH/DMF system are more favorable than those in the Diox/MeOH/DMF system. Overall, the Diox/EtOH/DMF system supports growth and coupling even more favorably than the Diox/MeOH/DMF system, confirming that solvent composition regulates chain rearrangement and thus the final nanowire morphology.

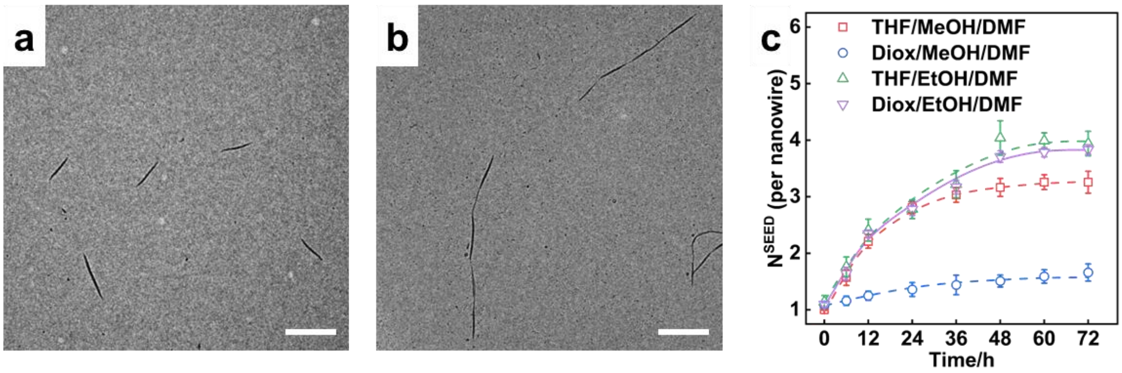

Supplementary Figure 17. Segmented nanowires prepared in Diox/EtOH/DMF system. (a) TEM image of the seed micelles prepared in Diox/EtOH mixture solution. (b) TEM image of the segmented nanowires prepared in Diox/EtOH/DMF mixture solution after aging at 30 °C for 48 hours. Scale bars: 1 μm. (c) Temporal variation of the number of seeds (N^{SEED}) for the Diox/EtOH/DMF system, and the corresponding temporal variations for the other solvent conditions are also plotted for comparison. Error bars represent mean ± standard deviation, $n \geq 200$.

Such a solvent-dependent behavior originates from the LC ordering of the PBLG core-forming blocks. Supplementary Table 1 summarizes the Hansen solubility parameters of PBLG, PNIPAM, and the solvents used. Because the end-to-end reactivity is dictated by how readily

PBLG chains can rearrange at micelle edges, the solvation environment plays a decisive role. In selective alcohols, methanol ($\delta = 30.20$ MPa$^{1/2}$) solvates PBLG more strongly than ethanol ($\delta = 26.60$ MPa$^{1/2}$). The weaker solvation of PBLG in ethanol introduces a mild solvophobic drive that facilitates chain mobility and LC reorganization. Consequently, growth and end-to-end coupling occur more efficiently in the THF/EtOH/DMF mixture, consistent with our experimental results. By explicitly incorporating these solubility considerations, we clarify how solvent identity controls segment rearrangement and thus the kinetics of fusion and coupling. These analyses strengthen the molecular-level foundation of the proposed mechanism.

Supplementary Table 1. Hansen solubility parameters of PBLG, PNIPAM, and solvents

Sample	Hansen solubility parameters δ / MPa$^{1/2}$
PBLG	23.40
PNIPAM	23.50
DMF	24.90
THF	19.50
Diox	20.47
MeOH	30.20
EtOH	26.60

1.14 Effect of the incubation temperature

Temperature also modulates chain mobility within both the LC core (PBLG) and corona (PNIPAM), thereby regulating edge reactivity. Supplementary Figure 18a and Supplementary Figure 18b show the TEM images of the aggregates after incubating at 20 °C and 40 °C for 48 hours, respectively. At 20 °C, the LC blocks possess insufficient mobility. Chain rearrangement is strongly hindered, and only one or two segments form even after 48 h (Supplementary Figure 18a). At 40 °C, PBLG mobility increases, but PNIPAM chains collapse more tightly around the core, paradoxically restricting PBLG rearrangement and suppressing growth and coupling (Supplementary Figure 18b). At 30 °C, both PBLG and PNIPAM retain adequate mobility. This balanced condition uniquely permits efficient fusion growth followed by controlled end-to-end coupling, as confirmed by the evolution of N^{SEED} (Supplementary Figure 18c). These results demonstrate that precise thermal control is critical for enabling the LC-mediated rearrangement required for segment fusion.

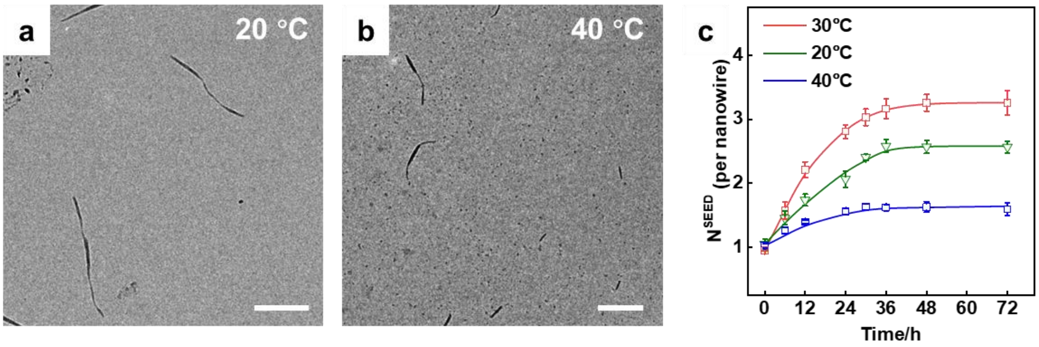

Supplementary Figure 18. TEM images of the aggregates after incubating at (a) 20 °C and (b) 40 °C for 48 hours. (c) Variations of the seed number N^{SEED} for the segmented nanowires formed at various temperatures. Error bars represent mean ± standard deviation, $n \geq 200$. Scale bars: 1 μm.

2. Theoretical Simulations

2.1 Brownian dynamics simulation method

The Brownian dynamics (BD) simulation was developed by Grest and Kremer [S6,S7], which is an efficient method for simulating polymer self-assembly. The BD simulation can well realize the implicit description of the continuum solvent and the simplification of internal motions. The temporal evolution of the beads is described by the pairwise potentials, the friction term, and the noise term. The above content obeys the Langevin equation in BD simulations. The beads are coupled to a heat bath, and the equation of motion is written as

$$m_i \frac{d^2\mathbf{r}_i}{dt^2} = \mathbf{F}_i - \Gamma_0 \frac{d\mathbf{r}_i}{dt} + \mathbf{W}_i(t) \tag{S-6}$$

where m_i is the mass of the i-th bead, Γ_0 is the friction constant, and $\mathbf{F}_i$ is the force acting on the i-th bead, which can be obtained by the derivatives of U_{mol} and U_{ij}. $\mathbf{W}_i(t)$ can be calculated through the fluctuation-dissipation relation

$$\left\langle \mathbf{W}_i(t) \cdot \mathbf{W}_j(t') \right\rangle = 6k_B T \Gamma_0 \delta_{ij} \delta(t-t') \tag{S-7}$$

In the simulation, periodic boundary conditions are imposed. The integration time step Δt = 0.005 τ, and the simulation temperature $k_BT = 3.0\varepsilon$ was selected [S8-S10]. Here, $\tau = (m\sigma^2/\varepsilon)^{1/2}$ is the time unit, m is the unit of mass, σ is the unit of length, and ε is the unit of energy.

We considered a simulation system consisting of rod-coil block copolymers in implicit solvents. The copolymers were modeled as linear chains with **R** beads of rod block and **C** beads of coil block, denoted by $\mathbf{R_mC_n}$ (the subscripts m and n denote the bead number of each block), as shown in Supplementary Figure 19. In the coarse-grained copolymer model, one bead

represents a cluster of atoms or repeat units. The coarse-grained model was parameterized based on experiments. By mapping multiple real atoms into an interaction site, the model can correspond to a block copolymer chain [S11].

The bead-bead interactions are given by the bonding potential U_{mol} and the nonbonding potential U_{ij}. The U_{mol} can maintain a desired molecular chain structure, while the U_{ij} describes the nonbonding interactions. For the rod blocks, the U_{mol} can be divided into two parts, that is, the bond stretching potential U_{bond} and the angle bending potential U_{angle}. All the neighboring beads are connected by a bond modeling with a harmonic spring potential, given by

$$U_{bond}(r) = \frac{1}{2} k_b (r - r_0)^2 \tag{S-8}$$

where k_b is the bond spring constant, r is the distance between the chemically bonded beads, and r_0 is the equilibrium bond length. To realize the rigidity of the rod blocks, the angle bending potential is introduced

$$U_{angle}(\theta) = \frac{1}{2} k_a (\theta - \theta_0)^2 \tag{S-9}$$

where k_a is the angle spring constant, θ is the angle between two neighboring bonds, and θ_0 is the equilibrium angle.

The nonbonding potential U_{ij} between any pair of i-th and j-th beads is given by the standard Lennard-Jones (LJ) potential

$$U_{ij} = \begin{cases} 4\varepsilon_{ij}\left[\left(\frac{\sigma}{r_{ij}}\right)^{12} - \left(\frac{\sigma}{r_{ij}}\right)^{6} - \left(\frac{\sigma}{r_{ij}^{c}}\right)^{12} + \left(\frac{\sigma}{r_{ij}^{c}}\right)^{6}\right], & r \le r_{ij}^{c} \\ 0, & r > r_{ij}^{c} \end{cases} \tag{S-10}$$

where $r_{ij} = \left|\mathbf{r}_i - \mathbf{r}_j\right|$ with $\mathbf{r}_i$ and $\mathbf{r}_j$ being the positions of the *i*-th and *j*-th beads, respectively. r_{ij}^c is the cutoff distance, and ε_{ij} is the interaction parameter between the *i*-th and *j*-th beads.

The simulations were performed by applying the simulator, a coarse-grained molecular dynamics program based on LAMMPS [S12].

2.2 Coarse-grained model and parameter settings

Bead number. The bead number was chosen appropriately to match the experimental data. For the PBLG-*b*-PNIPAM block copolymer used in this work (Supplementary Figure 19a), the degree of polymerization for the PBLG block and PEG block is $N_{PBLG} = 237$ and $N_{PNIPAM} = 118$, respectively. The PBLG chains are rod blocks with α-helix conformation, and the PNIPAM chains are coil blocks. The rod-coil block ratio is about 2:1. To capture the essential features of the PBLG-*b*-PNIPAM block copolymer molecule, we constructed a BD model of the rod-coil block copolymer in implicit solvents. The copolymers were modeled as linear chains with R beads of rod block (green) and C beads of coil block (red). In the BD simulations, to maintain the rod block of rigidity, the length of rod blocks cannot be too short. On the other hand, due to the limitation of computing resources, the length of rod-coil copolymers cannot be too long. Therefore, a moderate bead number for each R block (N_R= 6) and each C block (N_c= 3) was chosen. Thus, a coarse-grained model R_6C_3 was constructed (Supplementary Figure 19b), where one R or C bead represents 40 repeating units for the PBLG or PNIPAM blocks.

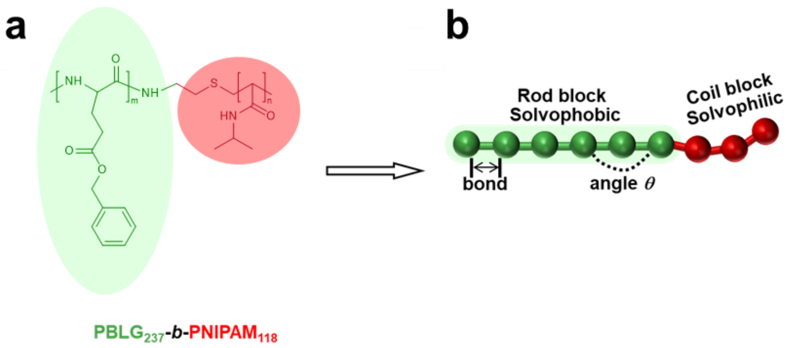

Supplementary Figure 19. Coarse-grained model of $PBLG_{237}$-*b*-$PNIPAM_{118}$. (a) $PBLG_{237}$-*b*-$PNIPAM_{118}$ block copolymer. (b) The coarse-grained model of an amphiphilic rod-coil copolymer $\mathbf{R_6C_3}$.

Bond and angle. All the neighboring beads were connected by a bond modeling with a harmonic spring potential, which was given by Supplementary Eqn. (8). The equilibrium bond length r_0 was 1.0σ for the rod and coil blocks. The value of k_b was set as 100 ε/σ^2 to avoid the over-stretching of bonds. To model the rigidity of rod blocks, the angle bending potential was introduced by Supplementary Eqn. (9). In the simulations, the angle bending potential was employed by setting the angle spring constant $k_a = 100\varepsilon$ and $\theta_0 = 180°$ for the rod block, while for the coil block, $k_a = 0$.

Cutoff radius. To take account of the solvent selectivity, the cutoff distance rules of the LJ potential Supplementary Eqn. (10) were defined as follows. For the hydrophilic coil blocks, the **C-C** interactions were effectively repelled to each other by setting r^c_{CC} as $2^{1/6}\sigma$. While the **R-R** interactions were effectively attracted to each other by setting r^c_{RR} as 2.5σ. To simulate the immiscibility between the rod and coil blocks, r^c_{RC} was set as $2^{1/6}\sigma$.

Interaction parameters and simulation time. To simulate **R-C** and **C-C** interactions, the pairwise interaction parameters were set as follows, $\varepsilon_{\mathrm{RC}} = \varepsilon_{\mathrm{CC}} = 1.0\varepsilon$. The higher hydrophobicity was simulated by the stronger interaction strength $\varepsilon_{\mathrm{RR}}$ between the **R** and **R** beads. For the construction of cylindrical seeds, the aggregates were self-assembled from the $\mathbf{R_6C_3}$ copolymers at the interaction parameters $\varepsilon_{\mathrm{RR}}$ of 2.7ε. BD simulations with 1.0×10^8 steps ($5.0\times10^5\tau$) were carried out so that the simulation time was long enough for the system to achieve an equilibrium state. In the seeded-growth process, the copolymer chains were put into a simulation box containing the cylindrical seeds. The interaction parameter $\varepsilon_{\mathrm{RR}}$ was 2.7ε. BD

simulations with 2.0×10^7 steps ($1.0\times10^5\,\tau$) were chosen to observe the growth process, which is long enough for the system to reach equilibrium.

Simulation box. All the simulations were performed in a cubic cell using a dynamic algorithm with the temperature-controlling method (NVT ensemble). For the construction of cylindrical seeds, the box size was set as $60\times60\times60\ \sigma^3$. In the seeded-growth stage, the simulation box was set as $200\times200\times200\ \sigma^3$.

2.3 Growth and coupling behavior of cylindrical micelles

When the interaction parameter ε_{RR} was 2.7ε, we observed coupling behavior in the grown cylindrical micelles. Specifically, it shows that the rigid chain blocks at both ends of the micelles and the coupling region tended to adopt a cholesteric LC-like arrangement (see the orange marked region in Supplementary Figure 20).

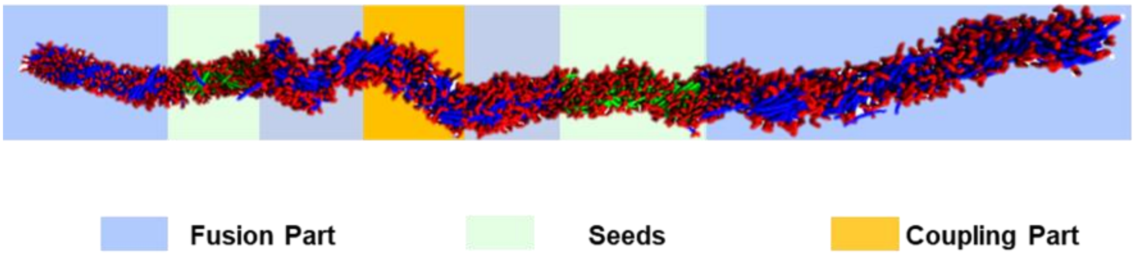

Supplementary Figure 20. The coupling behavior of cylindrical micelles.

Supplementary Figure 21a displays a typical cylindrical micelle and the definitions of orientation angles for the orientation of the rod blocks. Supplementary Figure 21b displays typical small aggregates self-assembled from the added block copolymers. The newly formed aggregates do not have perfect cholesteric LC cores. When these aggregates fuse with seeds, fusion can eliminate the unfavorable exposed surface, and the rod blocks in the small aggregates gradually rearrange. Supplementary Figure 21c shows the variations of the orientation angles for the rod blocks in the growing cylindrical micelles. The orientation of the rod blocks can be adjusted to match the cholesteric LC structure in the cores of the seeds to generate the ordered packing manner in the grown micelles.

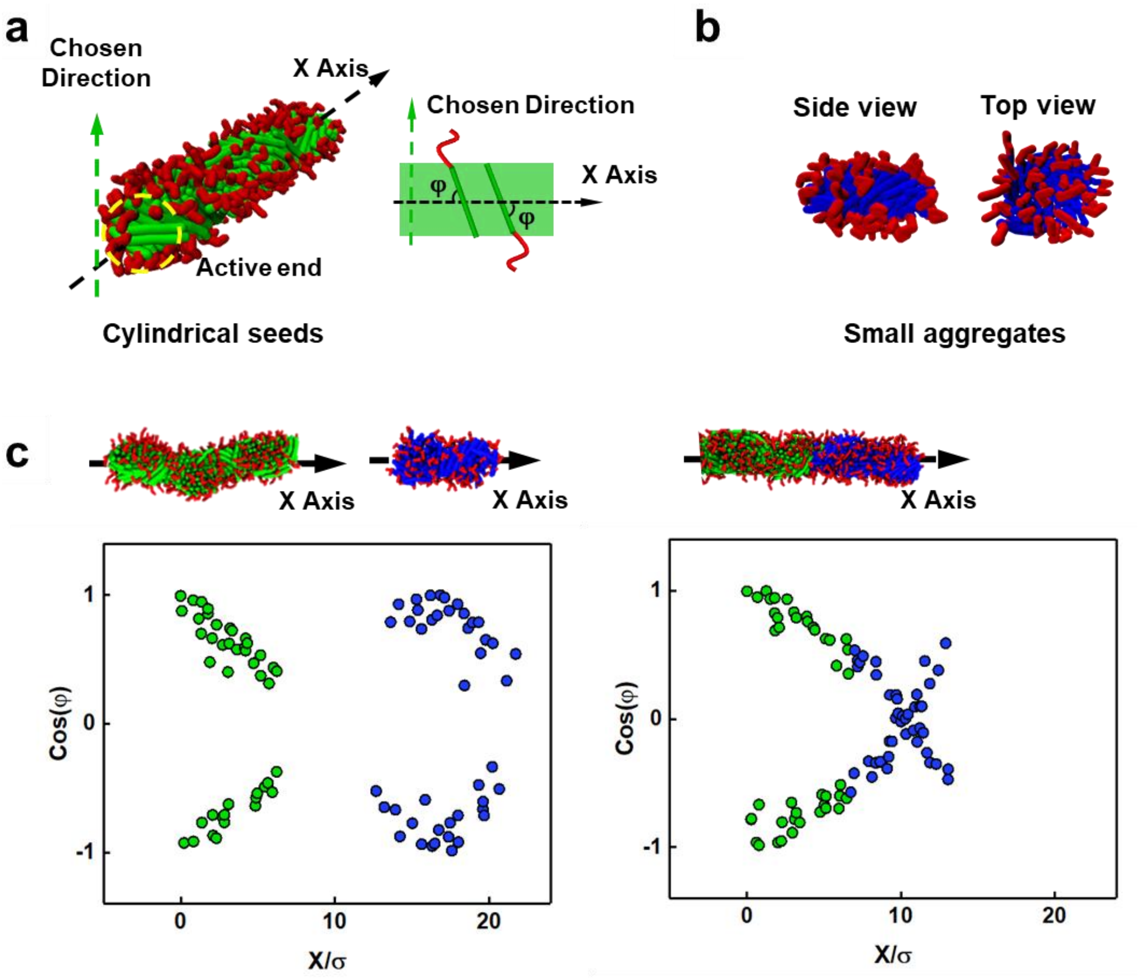

Supplementary Figure 21. Structural details and chain rearrangements of the growing cylindrical micelles. Typical structures of (a) cylindrical seeds and (b) small aggregates. (c) Variations in the cosine of φ along the X-axis of the growing cylindrical micelles.

The simulation times required for the chain rearrangements during fusion growth and during end-to-end coupling were compared. As shown in Supplementary Figure 22, the fusion growth between a seed and a small aggregate takes *ca.* 1500 τ for the chains to rearrange (Supplementary Figure 22a). In contrast, the coupling of two grown cylindrical micelles takes about 5500 τ (Supplementary Figure 22b). This confirms that chain rearrangement in the coupling step is indeed much slower than in fusion growth.

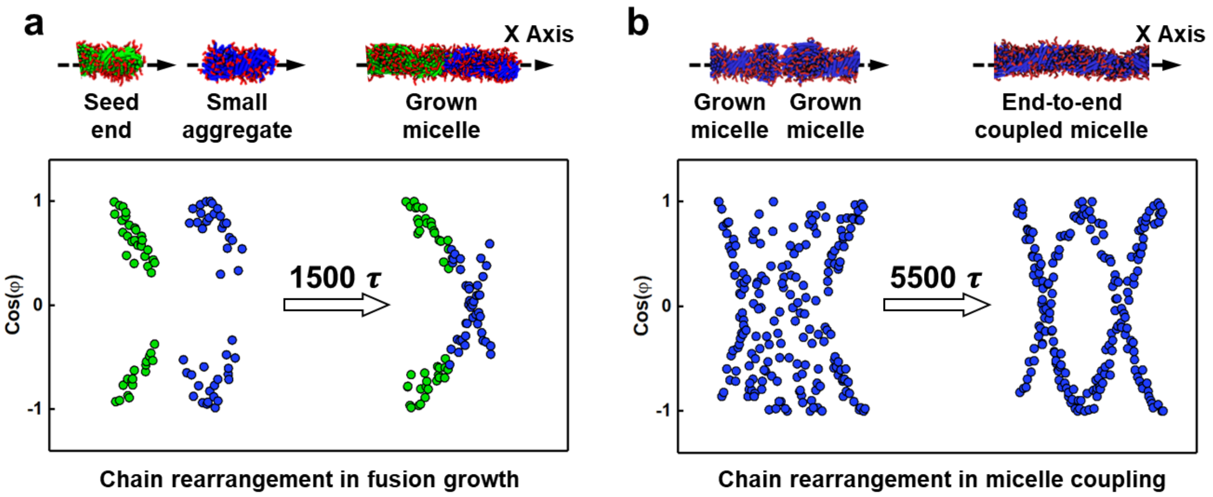

Supplementary Figure 22. Comparison of the chain rearrangements for fusion growth and end-to-end coupling. The consumed simulation time for chain rearrangements: (a) fusion growth of seed and small aggregate, (b) coupling of two contacted grown cylindrical micelles, where τ is the unit of simulation time.

2.4 Effect of interaction parameter on growth and coupling behaviors

Here, we increased the interaction parameter ε_{RR} in the simulations, which corresponds to the enhanced interaction of the PBLG blocks at high methanol content in the experiments. When the ε_{RR} is increased to 2.8ε (corresponding to higher methanol content), the fusion growth and end-to-end coupling can still be observed. However, the chain packing manners in the formed nanowires are relatively less disordered.

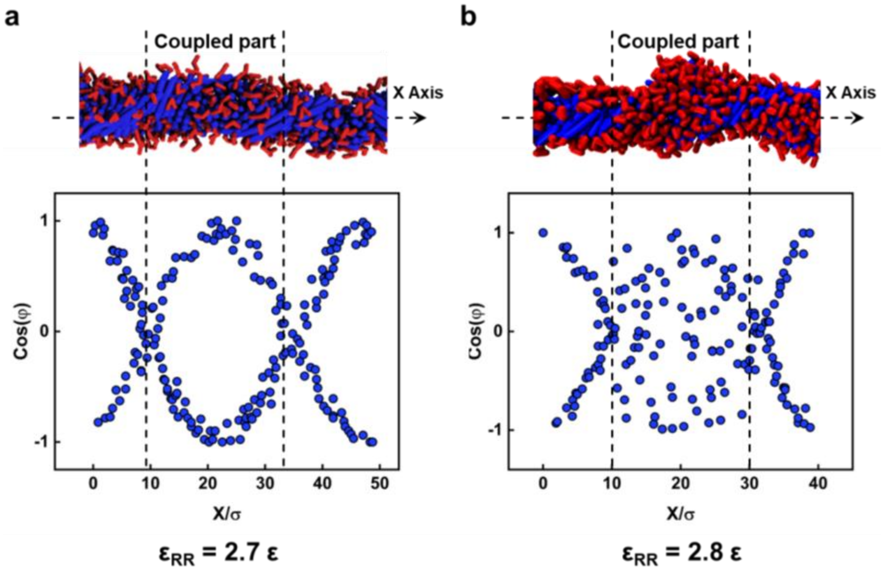

Supplementary Figure 23. Effect of interaction parameter on growth and coupling behaviors. Variations in the cosine of φ along the X-axis of the cylindrical micelles obtained at the ε_{RR} of (a) 2.7ε and (b) 2.8ε. Only the two ends of the grown micelles and their coupled parts are presented in the figures.

Supplementary Figure 23a shows the comparison of the variations in the cosine of orientation angles, φ, along the long axis of coupled micelles formed at 2.7ε and 2.8ε, respectively. At the interaction strength of 2.8ε, the LC chains within the ends of the grown micelles have an imperfect cholesteric LC arrangement, and the chains can not well rearrange themselves after coupling (see Supplementary Figure 23b). This result indicates that the

enhancement of the interaction parameter ε_{RR} (higher methanol content) makes the chain rearrangement more difficult and limits the fusion growth and coupling of micelles.

References

1. Zhuang, Z., Cai, C., Jiang, T., Lin, J. & Yang, C. Self-assembly behavior of rod–coil–rod polypeptide block copolymers. *Polymer* **55**, 602-610 (2014).

2. Blout, E. & Karlson, R. Polypeptides. III. The synthesis of high molecular weight poly-γ-benzyl-L-glutamates. *J. Am. Chem. Soc.* **78**, 941-946 (1956).

3. Tian, Z. Y., Zhang, Z., Wang, S. & Lu, H. A moisture-tolerant route to unprotected α/β-amino acid N-carboxyanhydrides and facile synthesis of hyperbranched polypeptides. *Nat. Commun.* **12**, 1-11 (2021).

4. Huang, C.-J. & Chang, F.-C. Polypeptide diblock copolymers: syntheses and properties of poly(*N*-isopropylacrylamide)-*b*-polylysine. *Macromolecules* **41**, 7041-7052 (2008).

5. Pantshwa, J. et al. Synthesis of novel amphiphilic poly(N-isopropylacrylamide)-*b*-poly(aspartic acid) nanomicelles for potential targeted chemotherapy in ovarian cancer. *J. Drug Delivery Sci. Technol.* **39**, 308-323 (2017).

6. Grest, G. S. & Kremer, K. Molecular dynamics simulation for polymers in the presence of a heat bath. *Phys. Rev. A Gen. Phys.* **33**, 3628-3631 (1986).

7. Grest, G. S., Lacasse, M. D., Kremer, K. & Gupta, A. M. Efficient continuum model for simulating polymer blends and copolymers. *J. Chem. Phys.* **105**, 10583-10594 (1996).

8. Ding, W., Lin, S., Lin, J. & Zhang, L. Effect of chain conformational change on micelle structures: experimental studies and molecular dynamics simulations. *J. Phys. Chem. B* **112**, 776-783 (2008).

9. Horsch, M. A., Zhang, Z. & Glotzer, S. C. Self-assembly of polymer-tethered nanorods. *Phys. Rev. Lett.* **95**, 056105 (2005).

10. Lin, S., Numasawa, N., Nose, T. & Lin, J. Brownian molecular dynamics simulation on self-assembly behavior of rod-coil diblock copolymers. *Macromolecules* **40**, 1684-1692 (2007).

11. Srinivas, G., Shelley, J. C., Nielsen, S. O., Discher, D. E. & Klein, M. L. Simulation of diblock copolymer self-assembly, using a coarse-grain model. *J. Phys. Chem. B* **108**, 8153-8160 (2004).

12. http://lammps.sandia.gov.
