# Supplementary material for: Supramolecular coupling of cylindrical micelles following seeded-growth
Source: Nat Commun. 2026 Feb 26;17:3247. doi: 10.1038/s41467-026-69785-3 (PMC13062008; doi:10.1038/s41467-026-69785-3)
Supplement: Supplementary file 2 — Description of Additional Supplementary Files [file 41467_2026_69785_MOESM2_ESM.pdf]

Description of Additional Supplementary Files

File Name: Supplementary Movie 1
Description: A video was made from the trajectories of computer simulations to visualize micelle growth and coupling behaviors.
